# Activities of Endochin-Like Quinolones Against *in vitro* Cultured *Besnoitia besnoiti* Tachyzoites

**DOI:** 10.3389/fvets.2020.00096

**Published:** 2020-02-26

**Authors:** Naja Eberhard, Vreni Balmer, Joachim Müller, Norbert Müller, Rolf Winter, Soviti Pou, Aaron Nilsen, Mike Riscoe, Samuel Francisco, Alexandre Leitao, J. Stone Doggett, Andrew Hemphill

**Affiliations:** ^1^Vetsuisse Faculty, Institute of Parasitology, University of Bern, Bern, Switzerland; ^2^VA Portland Health Care System Research and Development Service, Portland, OR, United States; ^3^Faculdade de Medicina Veterinária, CIISA—Centro de Investigação Interdisciplinar em Sanidade Animal, Universidade de Lisboa, Lisbon, Portugal

**Keywords:** *Besnoitia besnoiti*, endochin-like quinolones, SAR, electron microscopy, mitochondrial inhibition

## Abstract

Endochin-like quinolones (ELQs) potently inhibit the proliferation of *Plasmodium, Toxoplasma, Neospora*, and *Babesia* by targeting the cytochrome *b* Qo and Qi sites and interfering with oxidative phosphorylation and pyrimidine biosynthesis. The activities of 14 different ELQs were assessed against *B. besnoiti* tachyzoites grown in human foreskin fibroblasts (HFF) by quantitative real time PCR. The values for 50% proliferation inhibition (IC50) of five ELQs were determined in a 3-days growth assay after an initial screen of 12 ELQs at 0.01, 0.1, and 1 μM. The IC50s of ELQ-121, -136, and -316 were 0.49, 2.36, and 7.97 nM, respectively. The IC50s of ELQs tested against *B. besnoiti* were higher than IC50s previously observed for *P. falciparum* and *T. gondii*. However, the *B. besnoiti* cytochrome *b* sequence and the predicted Qo and Qi ELQ binding sites in the *Toxoplasma, Neospora*, and *Besnoitia* cytochrome *b* are virtually identical, suggesting that the differences in ELQ susceptibility are not due to variations in the substrate binding sites. TEM of ELQ-treated parasites primarily demonstrated alterations within the parasite mitochondrion, profound thickening of the nuclear membrane, as well as increased vacuolization within the tachyzoite cytoplasm. Long-term treatment assays of intracellular *B. besnoiti* with ELQs for up to 20 days followed by the release of drug pressure caused a substantial delay in parasite growth and proliferation while ELQs were present, but parasite proliferation resumed days after ELQs were removed. Interestingly, structural alterations persisted after ELQ removal and parasite proliferation was slowed. These findings provide a basis for further *in vivo* studies of ELQs as therapeutic options against *B. besnoiti* infection.

## Introduction

*Besnoitia besnoiti* is a cyst-forming coccidian parasite (1), which is closely related to *Neospora caninum* and *Toxoplasma gondii* and is the causative agent of bovine besnoitiosis (2, 3). Bovine besnoitiosis was originally restricted to endemic areas in Portugal, Spain, and South of France (4). Lately, new outbreaks of the disease have been reported in other parts of Europe, including Germany, Italy, Hungary, Croatia, Belgium, and Switzerland (2, 5). Due to the geographic expansion and increased number of cases, the European Food Safety Authority (EFSA) has proclaimed besnoitiosis as a re-emerging disease (6, 7).

Similar to other cyst-forming coccidian parasites, *B. besnoiti* is suspected to have an indirect life cycle in which a carnivore represents the definitive host and cattle as well as wild bovids serve as intermediate hosts. Although cats have been shown to be the definitive host of several other *Besnoitia* species (*B. darlingi, B. wallacei, B. orictofelisi*, and *B. neotomofelis*), the definitive host of *B. besnoiti* has not been identified (1).

Bovine besnoitiosis induces serious economic losses in terms of reduced milk and meat production, infertility and sterility in bulls, skin damage, poor body conditions, occasional abortions in dams, premature slaughter, and deaths (2, 4). The formation of dermal tissue cysts at the chronic stage is a hallmark of bovine besnoitiosis, and causes dermal lesions, hair loss, hyperkeratosis, hyperpigmentation and rather dramatic thickening, hardening and folding or wrinkling of the skin, especially around the neck, shoulders and rump. To date, none of the members of the genus *Besnoitia* has been shown to infect humans (3).

The treatment options to tackle bovine besnoitiosis are limited. The disease could be controlled by either culling an infected animal or possibly prevented by vaccination and/or chemotherapy (8). However, no vaccine against *B. besnoiti* is licensed in Europe, and there are no effective drugs available to date (6). Since several aspects of the biology of *B. besnoiti* are not well-understood, targeted drug development against this disease is far from becoming reality. Thus, repurposing of compounds that are already licensed or under development is a faster way to identify novel candidate therapeutics against *B. besnoiti* infection. This has been demonstrated in studies on thiazolides (9), cationic arylimidamides (10), bumped kinase inhibitors (BKIs), diclazuril and decoquinate (11, 12), and buparvaquone (13).

Endochin-like quinolones (ELQs) are derived from endochin, a 4(1H)-quinolone that is a cytochrome *bc*_1_ complex inhibitor, similar to atovaquone, or buparvaquone. Inhibitors of the mitochondrial electron transport chain can bind to the oxidative Qo site or the reducing Qi site of cytochrome *b*, prevent oxidative phosphorylation and cause deprivation of essential molecules and the generation of reactive oxygen species (ROS) (14). Disruption of the mitochondrial electron transport chain of parasites has devastating consequences, and has been the focus of the development of anti-parasitic drugs for decades (15). Endochin had been shown to exhibit promising activity against experimental avian malaria (16), but was unstable when exposed to murine, rat, and human microsomes, prohibiting its clinical use. However, ELQs with aromatic side chains at position three were shown to exhibit IC50s in the range of 0.1 nM against *Plasmodium falciparum in vitro* (17). Doggett et al. (18) and Nilsen et al. (19) reported the synthesis of novel endochin analogs with a diphenylether side chain at the third position, and various substitutions at the quinolone ring at position 5, 6, or 7. These include ELQ-300 and ELQ-316, which confer Qi site inhibition, and ELQ-400 (20), which has been reported to target both Qo and Qi sites of cytochrome *b* of *S. cerevisiae* and potentially other organisms (21). ELQ-316 inhibited *T. gondii* proliferation *in vitro* at sub-nanomolar concentrations. The drug was highly effective in a murine acute disease model, and reduced the *Toxoplasma* cyst burden in a model of latent infection by 76–80% (18, 22). In order to improve the bioavailability and water-solubility of ELQs, ethyl carbonate prodrugs were synthesized (23). ELQ-334 is such a prodrug of ELQ-316 that is much better absorbed and yields a 6-fold increase in ELQ-316 exposure after oral uptake and metabolization. More recently, a series of ELQs were reported to exhibit promising *in vitro* activity against *N. caninum* tachyzoites (24). ELQ-400 treatment in mice experimentally infected with *N. caninum* tachyzoites lead to significantly reduced parasite burden (25). ELQ-334 treatment of *N. caninum* infected pregnant mice did not reduce the cerebral parasite burden in the dams, but inhibited vertical transmission and clinical signs in newborn pups and in the dams (24). The experimental efficacy of ELQs as well as the fact that they have been optimized to avoid targeting the human cytochrome *bc*_1_ complex renders them promising anti-parasitic compounds (26).

In this study, we assessed the *in vitro* activities of 12 distinct ELQ analogs against the *B. besnoiti* strain *Bb* Lisbon. The most active ELQs against this parasite (ELQ-121 and ELQ-136), as well as ELQ-316 and its prodrug ELQ-334 were studied for their parasiticidal vs. parasitostatic activities, and ultrastructural alterations induced by these compounds within *B. besnoiti* tachyzoites were investigated by transmission electron microscopy (TEM).

## Materials and Methods

### Tissue Culture Media, Biochemicals, and Drugs

If not stated otherwise, all tissue culture media were purchased from Gibco-BRL (Zürich, Switzerland), and biochemical reagents were from Sigma (St. Louis, MO). ELQs were obtained from VA Portland Healthcare System Department of Research and Development Service in Portland, Oregon, USA, and were synthesized as previously described (22). For *in vitro* studies the compounds were stored as 10 mM stock solutions in DMSO at −20°C.

### Host Cell Cultivation and Parasite Cultures

Human foreskin fibroblasts (HFF) were maintained as described earlier (24, 25). The *B. besnoiti* isolate *Bb* Lisbon was obtained, following published procedures (27), from a naturally occurring chronic case of bovine besnoitiosis. *Bb* Lisbon tachyzoites were maintained by serial passages in HFF in culture medium (DMEM containing 10% FCS). Tachyzoites were harvested by removal of the cell layer with a cell scraper, repeated passage through a 25 G needle, and Sephadex G25 chromatography as described (13).

### Primary *in vitro* Screening

In a primary screen, a small library of 14 ELQs (Table 1) was assessed for their effects on *in vitro* proliferation of *Bb* Lisbon tachyzoites. Six-well tissue culture plates were seeded with 1.2 × 10^5^ HFF per well and were incubated for 3 days at 37°C and 5% CO_2_. Prior to infection of confluent monolayers, the 10 mM compound stock solutions, as well as DMEM/10% FCS used for the dilution, were heated up to 37°C to optimize compound solubility. Serial dilutions of the compounds (1, 0.1, and 0.01 μM, all in duplicate) were prepared in DMEM and added to the confluent HFF monolayers. Controls received DMSO diluted in culture medium corresponding to the highest drug concentration. The medium containing these serially diluted drugs were added to HFF monolayer, and immediately thereafter cultures were infected with freshly isolated *Bb* Lisbon tachyzoites (2.5 × 10^4^ tachyzoites/well). Infected cells were cultured at 37°C, 5% CO_2_ for 3 days. Subsequently, cells were collected with a cell scraper, centrifuged and resuspended in 1 ml PBS. DNA purification was performed according the protocol of the MACHINERY-NAGEL Rapid Lyse DNA isolation Kit. The DNA was eluted in 100 μl elution buffer, and specimens were stored at −20°C. Drug activity assessments for each drug concentration were done in duplicate, and each assay was done at least twice. *B. besnoiti* parasite load was quantified by RT-PCR as described earlier (13). The relative *B. besnoiti* growth values at the three drug concentrations in relation to untreated control cultures are summarized in Table 2.

**Table 1:** Structures of the 14 ELQ compounds and IC_50_ values measured against *Besnoitia besnoiti, Neospora caninum* and *Toxoplasma gondii* tachyzoites.

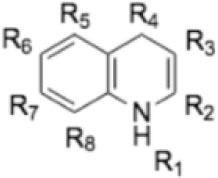									
**ELQ**	**MW (g/mol)**	**R3**	**R4**	**R5**	**R6**	**R7**	***Bb*****IC**_**50**_ **(nM)**	***Nc*****IC**_**50**_ **(nM)^a^**	***Tg*****IC**_**50**_ **(nM)^c^**
100	287.4	-C_7_H_15_ 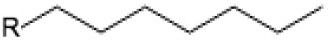	=O	/	/	-OCH_3_	ND	0.75	0.07
121	293.3	-C_7_H_15_ 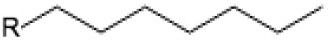	=O	-F	/	-F	0.495	0.08	0.006
127	257.0	-C_7_H_15_ 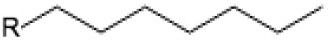	=O	/	/	/	ND	2.45	2.8
136	275.4	-C_7_H_15_ 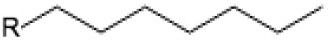	=O	-F	/	/	2.634	0.02	0.13
271	411.0	-C_13_H_8_O_2_F_3_ 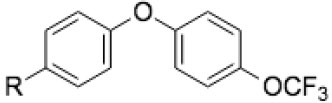	=O	/	/	/	ND	2.75	5.6
300	475.8	-C_13_H_8_O_2_F_3_ 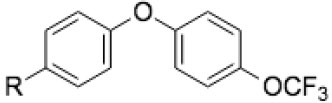	=O	/	-Cl	-OCH_3_	ND	0.33	25
316	459.4	-C_13_H_8_O_2_F_3_ 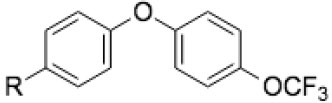	=O	/	-F	-OCH_3_	7.972	0.66	0.35
334	531.4	-C_13_H_8_O_2_F_3_ 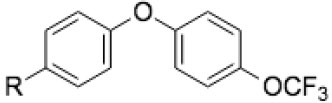	-O_3_C_3_H_5_ 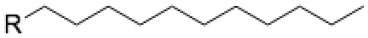	/	-F	-OCH_3_	5.81	3.33	ND
400	447.3	-C_13_H_8_O_2_F_3_ 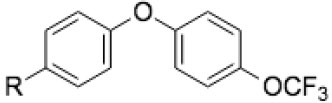	=O	-F	/	-F	72.2	4^b^	5
433	305.4	-C_7_H_15_ 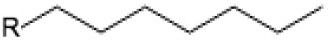	=O	/	-F	-OCH_3_	ND	4.67	3.8
434	377.9	-C_11_H_23_ 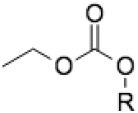	=O	/	-Cl	-OCH_3_	ND	3.81	11
435	361.5	-C_11_H_23_ 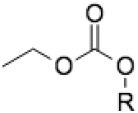	=O	/	-F	-OCH_3_	ND	2.01	0.24
436	313.5	-C_11_H_23_ 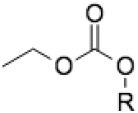	=O	/	/	/	ND	1.04	0.68
437	349.5	-C_11_H_23_ 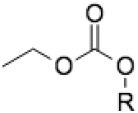	=O	-F	/	-F	ND	0.09	0.003

a *Results reported in Anghel et al. (24)*;

b *reported in Müller et al. (25)*;

c *results reported in McConnell et al. (22), R, endochin core; ND, not done*.

**Table 2:** Primary assessment of ELQ activities in terms of growth inhibition of B. besnoiti tachyzoites.

**ELQ**	**100**	**121**	**127**	**136**	**271**	**300**	**316**	**334**	**400**	**433**	**434**	**435**	**436**	**437**
%growth at 0.01 μM	100	**10**	100	**11**	100	100	**42**	**45**	**24**	12	70	62	74	21
%growth at 0.1 μM	93	**0**	10	**0**	21	67	**12**	**7**	**0**	5	ND	9	6	0
%growth at 1 μM	3	**0**	0	**0**	1	8	**5**	**0**	**0**	0	25	8	0	0

*Cultures were exposed to drugs at 0.01, 0.1, and 1 μM during 3 days, and parasite proliferation was quantified by RT-PCR. ELQ-121, -136, -316, -334, and -400 (indicated in bold) were selected for IC_50_ determinations. For information on the compound structures please refer to Table 1*.

### Secondary Screening: Determination of the Half-Minimal Inhibitory Concentration (IC50)

The IC50 determinations for ELQ-121, -136, -316, -334, and -400 in *B. besnoiti* infected HFF were done using the identical setup as for the primary screening (see above). Culture media containing compounds ELQ 121 and 136 were prepared at the following concentrations: 50, 10, 5, 1, 0.5, 0.1, 0.05, 0.01, and 0.001 nM. Serial dilutions of the compounds ELQ-316, -334, and -400 were done at 25, 5, 0.75, 0.25, 0.1, 0.05, 0.01, 0.005, and 0.001 μM. Controls were devoid of drug, but contained DMSO according to the highest drug concentration used. After 3 days of treatment, cells were collected with a cell scraper, centrifuged and resuspended in 1 ml PBS. DNA purification was done as described for the primary screening, and samples were stored at −20°C prior to RT-PCR quantification of parasite load (9, 13). The IC50 was determined by a logarithmic calculation (logit-log) of the relative growth (RG), with growth in the non-drug treated control set at 1. The logit-log-transformation formula ln[RG/(1-RG)] = a × ln(drug concentration) + b was accomplished and a subsequent regression analysis by an Excel software “Analysis Tool Pack” package was done (Microsoft, Seattle, WA).

### Transmission Electron Microscopy (TEM)

Confluent HFF grown in T25 tissue culture flasks were infected with 3 × 10^5^ *Bb*-Lisbon tachyzoites, maintained for 48 h at 37°C/5% CO_2_, treated with 2.5 μM ELQ-121, -136, -316, or -334 during 6, 12, 24, and 48 h as indicated in the text, and were fixed and processed for TEM analyses as previously described (13). Briefly, medium was discarded, and monolayers were fixed in 5 ml 2% glutaraldehyde in 0.1 M Na-cacodylate buffer (fixation solution) for 10 min at room temperature (RT), and carefully removed from the flask with a cell scraper. After centrifugation (10 min, 4°C, 1,200 rpm), fresh fixation solution was added over night at 4°C. The samples were washed in 0.1 M Na-Cacodylate-buffer, and 1 ml osmium tetroxide solution [2% in 0.1 M Na-Cacodylate buffer (pH 7.3)] was added for 1 h at RT. Samples were washed 3× with water and 50 μl UranyLess solution (Electron Microscopy Sciences, Hatfield, PA, USA) was added for 30 min at RT. Subsequently, samples were washed in water and were dehydrated by stepwise incubation in ethanol (30, 50, 70, 90, and 3 x 100%). They were embedded in three changes of EPON-812 resin, and polymerization was achieved at 60°C overnight. Ultrathin (80 nm) sections were cut using a Reichert and Jung ultra-microtome (Vienna, Austria), and were loaded onto 300-mesh formvar-carbon coated grids (Plano GmbH, Marburg, Germany). Finally, grids were contrasted using UranyLess and lead citrate solutions (EMS) according to the manufacturer's instructions. Samples were viewed on a FEI Tecnai Spirit BioTwin transmission electron microscope operating at 80 kV.

### Long-Term Treatment Assays

To investigate whether ELQs would affect an established infection of *B. besnoiti* in HFF and whether the drug would act parasiticidal or parasitostatic, long-term treatment assays were performed as described earlier for *N. caninum* and *T. gondii* (24, 28). Briefly, confluent HFF were infected with 5 × 10^5^ tachyzoites for 4.5 h, washed two times with HBSS (Hanks balanced salt solution), and were then exposed to 2.5 μM ELQ-121, -136, -316, or -334 for periods of either 3, 6, 9, 13, 15, 20, or 23 days, with medium changes and/or addition of fresh compound-containing medium every 3 days. Once the drug pressure was released, the cultures were inspected daily for a period of 3 up to 23 days, and micrographs were taken by light microscopy. In the case of ELQ-316, parasites exposed to this compound for 20 days, followed by culture without compound for 10 days, were processed for TEM as described above.

## Results

### Primary *in vitro* Screening

For a preliminary efficacy assessment, *B. besnoiti* infected HFF were exposed to the 14 ELQs at 1, 0.1, and 0.01 μM. Dose-dependent effects were seen for all compounds (Table 2). Maximum growth inhibition (100%) at 0.1 μM was observed for ELQ-121, ELQ-136, ELQ-400 and ELQ-437. However, while at 0.01 μM ELQ-437 and ELQ 400 treatments resulted in > 20% relative growth, the respective growth values for ELQ-121 and ELQ-136 were 10%, rendering those two drugs the most potent inhibitors of *B. besnoiti* proliferation.

ELQ-121 and ELQ-136 were selected for further investigations due to their potency in the primary screen. Based on previous results on ELQ efficacy against *T. gondii* and *N. caninum* (18, 24), we also selected ELQ-316 and its prodrug ELQ-334, as well as ELQ-400, for further studies. ELQ-400 has been shown to exhibit outstanding *in vivo* activity, better than atovaquone, in an acute toxoplasmosis mouse model (22), and there is evidence that it acts as a dual Qo and Qi site inhibitor (21).

### IC50 Determinations

The IC50 values of ELQ-121, -136, -316, -334, and ELQ-400 are shown in Table 1, in comparison to those obtained for *T. gondii* (22) and *N. caninum* (24). The lowest IC50 for *B. besnoiti*, and the only one that was below 1 nM, was obtained with ELQ-121 (0.49 nM) and for *T. gondii* and *N. caninum*, respective values were also clearly below 1 nM, albeit by a factor of 10 (*N. caninum*) and 100 (*T. gondii)* lower. ELQ-136 had an IC50 value of 2.63 nM in *B. besnoiti*, which was roughly 163 and 20 times higher compared to reported values for *N. caninum* and *T. gondii*, respectively. The IC50 value obtained for ELQ-316 (7.97 nM) was 12 and 22 times higher compared to *N. caninum* and *T. gondii*, respectively, and the IC50 value for ELQ-334 (5.81 nM) was in the same range than the reported value in *N. caninum*. The highest IC50 value, 72 nM, was measured for ELQ-400. Overall, the IC50 values measured for *B. besnoiti* were substantially higher than those previously reported for the closely related apicomplexans *N. caninum* and *T. gondii*. For ELQ-334 we observed remarkable differences in IC50 values ranging from 2 to 82 nM (data not shown). The assays were repeated several times, and we found that freshly prepared compound aliquots (frozen and thawed not more than once) exhibited pronounced higher activities compared to aliquots that were frozen and thawed several times (IC50s 56.5–82.1 nM). This is possibly due to crystallization and/or adherence to glass or plastic.

### Sequence Alignment of Cytochrome *b* Qo and Qi Sites of *B. besnoiti* and *T. gondii*

**Figure 1:**
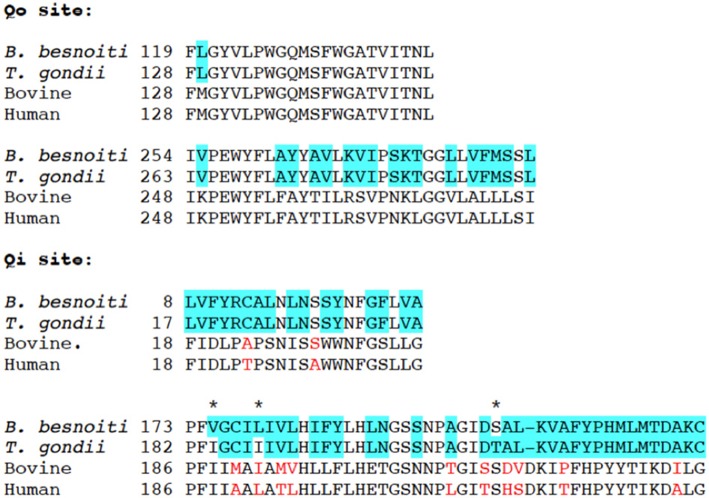
Alignment of *B. besnoitia, T. gondii*, bovine (*Bos Taurus*), and human mitochondrial cytochrome b sequences representing the Qo and Qi binding sites. Amino acids different in *B. besnoiti* and *T. gondii* are marked with an asterisk^*^, differences between *T. gondii/B. besnoiti* and *Bos taurus* are marked in blue, and the differences between bovine and human sequences are indicated in red font.

In order to investigate whether the differences in susceptibilities of *B. besnoiti* tachyzoites to the different ELQs could be related to differences in the putative cytochrome *b* Qo and Qi sites, the corresponding sequences of the *B. besnoiti* cytochrome *b* (Genbank Accession number PFH30612.1), *T. gondii* cytochrome *b* (XP_018634734.1), and bovine (AAZ95348.1) and human cytochrome *b* (UniProt P00156) were aligned according to McConnell et al. (22) and compared (Figure 1). The Qo binding site is identical in both parasite species, and the Qi site exhibits only 3 amino acid (aa) variations with amino acids that are similar to *T. gondii*. The *B. besnoiti* Qi site has a valine, leucine, and serine at position 175, 179, and 200, respectively, that correspond to isoleucine, isoleucine and threonine at the analogous positions in the *T. gondii* cytochrome *b*. Amino acids at these positions have not previously been found to influence substrate or inhibitor binding (29). These subtle changes are very unlikely to alter ELQ susceptibility. In addition, the *B. besnoiti* cytochrome *b* is 97% identical to the *T. gondii* cytochrome *b*, indicating that structural differences outside of the active sites are also very unlikely to influence ELQ binding. However, in comparison to the human enzyme, 18 aa variations are found in the parasite Qo sites, all but one in the N-terminal half of the binding site. More than 50 aa differences are found within the Qi sites of *B. besnoiti* and *T. gondii*, when compared to human and bovine cytochrome *b* Qi sites. These differences include aa that are involved in substrate and inhibitor binding and are likely determinants in ELQ specificity for apicomplexan pathogens (29).

### ELQ Treatments Induce Distinct Ultrastructural Changes in *B. besnoiti* Tachyzoites

*Besnoitia besnoiti* infected HFF were exposed to 2.5 μM ELQ-121, -136, -316, and -334 for different time spans, ranging from 6 to 48 h, or cells were maintained as corresponding DMSO controls. This concentration was chosen in order to exert a maximum drug pressure against the parasite without impairing the host cell integrity, similar to previous studies (24).

**Figure 2:**
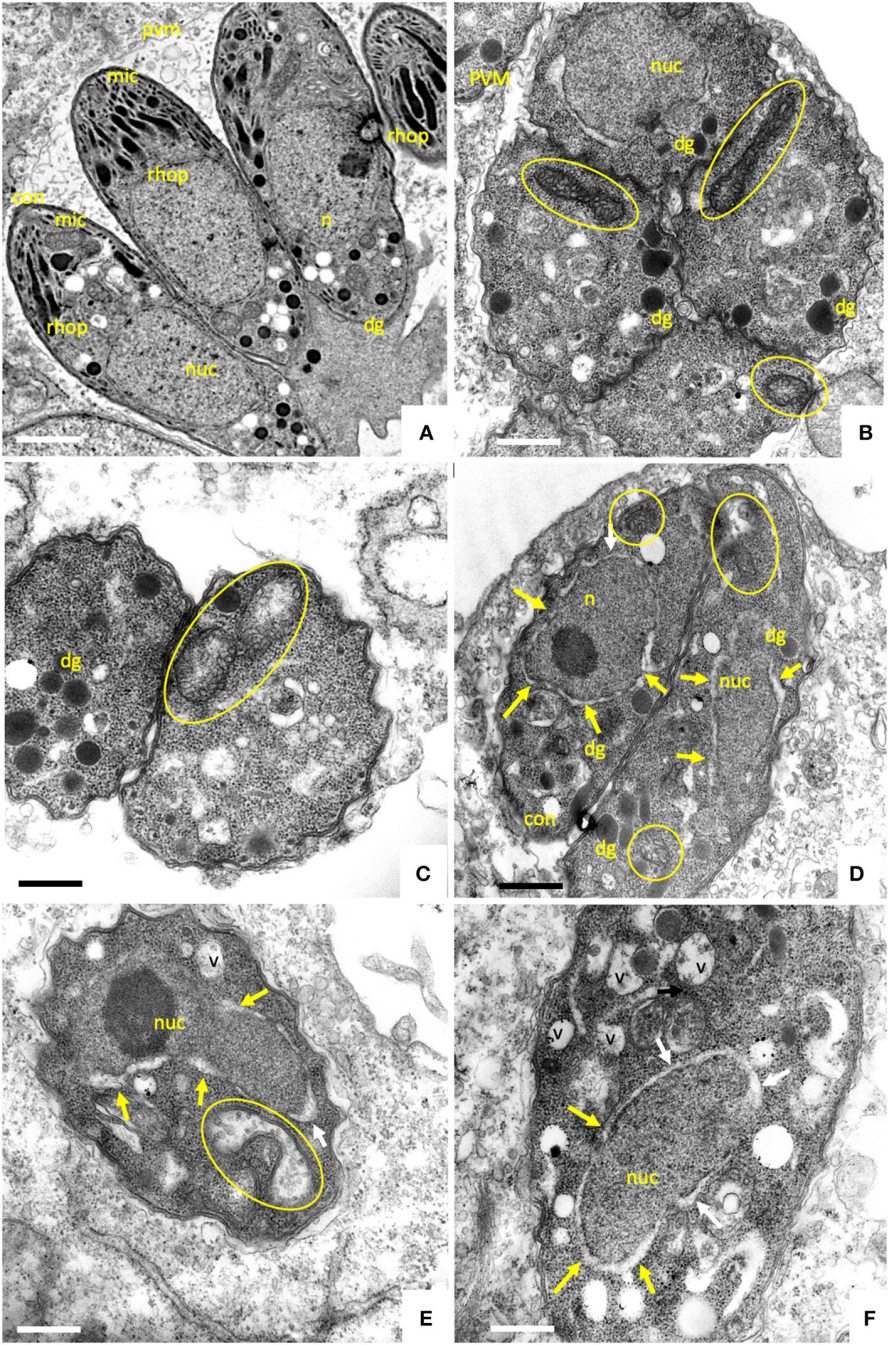
TEM of non-treated *B. besnoiti* tachyzoites cultured in human foreskin fibroblasts devoid of drugs at 48 h post-infection **(A**,**B)**. Tachyoites maintained in the presence of 2.5 μM ELQ-136 during 6 h are shown in **(C)**, parasites treated with ELQ-121 during 12, 24 and 48 h are shown in **(D–F)**, respectively. Encircled areas indicate the mitochondrion, white arrows point toward a thickening of the nuclear membrane; v, vacuole; nuc, nucleus; dg, dense granules; rhop, rhoptries; pvm, parasitophorous vacuole membrane; mic, micronemes; con, conoid. Bar in **(A)** = 500 nm; **(B**,**C)** = 300 nm; **(D)** = 400 nm; **(E**,**F)** = 300 nm.

Parasites in control cultures exhibited typical apicomplexan features (Figures 2A,B). The parasites were located intracellularly within a parasitophorous vacuole surrounded by a parasitophorous vacuole membrane (PVM), which separated parasites from the host cell cytoplasm. The typical hallmarks of apicomplexans, such as the apical complex including conoid, micronemes, rhoptries, and dense granule, were clearly visible. The single mitochondrion, parts of it visible in cross-sections, exhibited a clearly discernible electron dense matrix.

Upon treatment with 2.5 μM ELQ-136 and -121 for 6–12 h (Figures 2C,D), few alterations could be observed compared to control cultures. The central parts of the mitochondrial matrix became less electron dense in some ELQ-136 treated parasites, but cristae structures remained discernible (Figure 2C). However, the apical complex with conoid and micronemes remained unaffected. After 12 h of treatment with ELQ-121 (Figure 2D), the nuclear membrane became slightly thickened, widening the gap between nucleus and the surrounding cytoplasm. After 24 h ELQ-121 treatment (Figure 2E), the mitochondrion also started to exhibit distinct alterations, such as the loss of an electron dense matrix and associated cristae. In addition, as shown for ELQ-121 in Figure 2F, the entire nuclear membrane appeared thickened in many cells, and the number of cytoplasmic vesicles was increased. However, no further ultrastructural changes were evident, thus the typical apicomplexan secretory organelles such as rhoptries, micronemes, and dense granules, and the parasite plasma membrane and conoid, remained clearly discernible.

**Figure 3:**
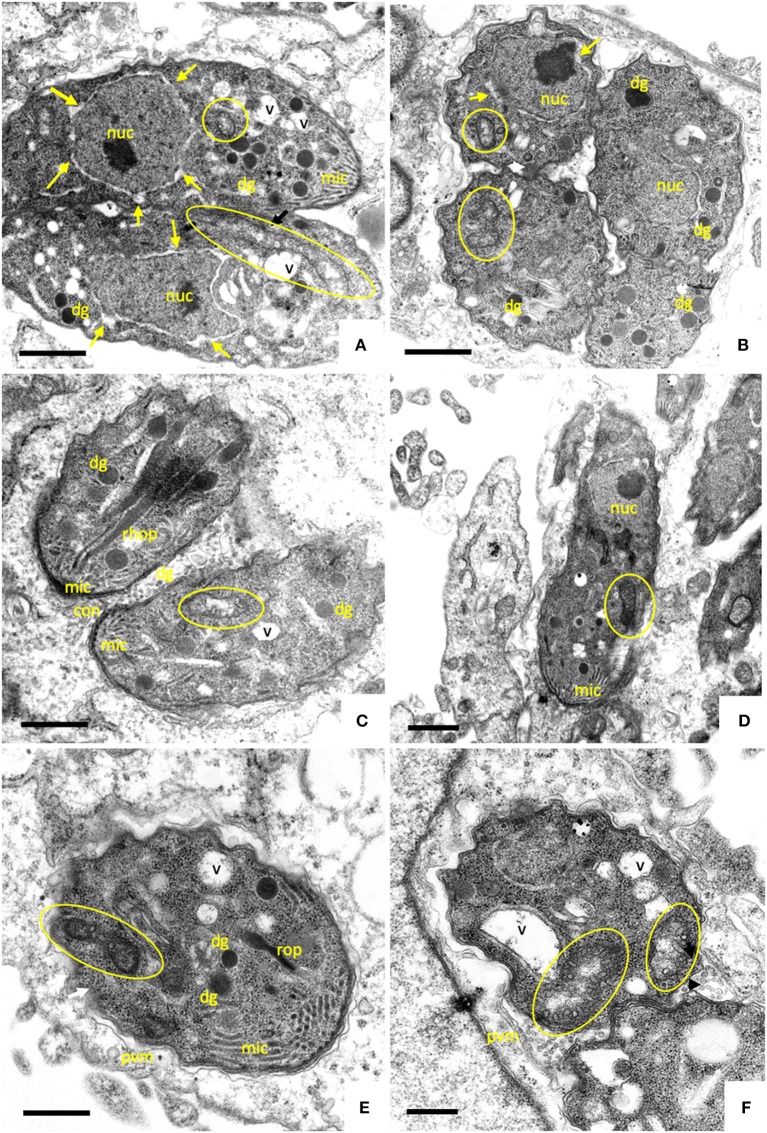
TEM of *B. besnoiti* infected HFF exposed to 2.5 μM ELQ-316 during 6, 24, and 48 h **(A–C)**, and ELQ-334 during 12, 24, and 48 h, respectively. The encircled areas indicate the mitochondrion, arrows indicate a thickening of the nuclear membrane; nuc, nucleus; dg, dense granules; rhop, rhoptries; mito, mitochondrion; PVM, parasitophorous vacuole membrane; mic, micronemes; con, conoid. Bars in **(A**,**B)** = 400 nm; **(C)** = 350 nm; **(D)** = 500 nm; **(E**,**F)** = 300 nm.

Upon treatment with 2.5 μM ELQ-316 (Figures 3A–C) for 6–12 h, alterations within the mitochondrion were also evident. The mitochondrial matrix became less electron dense already after 6 h. Like the other compounds, the drug also altered the spacing between the nucleus and cytoplasm, and vacuolization of the cytoplasm occurred after 6 h of treatment (Figure 3A). The apical complex with conoid and micronemes was not notably affected. Upon treatment with 2.5 μM ELQ-316 for 24 and 48 h (Figures 3B,C), alterations of the mitochondrion and vacuolization of the cytoplasmic were maintained. Other structures, however, remained unaffected. Exposure of *B. besnoiti* to 2.5 μM ELQ-334 for a time period of 12–24 h showed less pronounced alterations within the mitochondrion, and the apical complex and associated secretory organelles did not seem to be affected (Figures 3D,E). Upon ELQ-334 treatment over 48 h (Figure 3F), alterations in the mitochondrion became more evident, and cytoplasmic vacuolization clearly increased.

Overall, ELQ treatments most notably affected the tachyzoite mitochondrion by altering the electron dense matrix and cristae to the extent that the organelle, although exhibiting an intact mitochondrial membrane, appeared either devoid of content, or contained undefined residual structures of unknown composition. In general, parasites retained their shape and were still located inside a parasitophorous vacuole, surrounded by a PVM. The most clear and rapid mitochondrial alterations were found upon treatment with ELQ-136 and ELQ-316 (already at 6 h), whereas the treatment with its prodrug ELQ-334 changed the mitochondrial ultrastructure only after 24–48 h, most likely reflecting the time it takes for ELQ-334 to be converted to ELQ-316.

### Long-Term Treatments Reveal Parasitostatic Rather Than Parasiticidal Activities of ELQs

**Figure 4:**
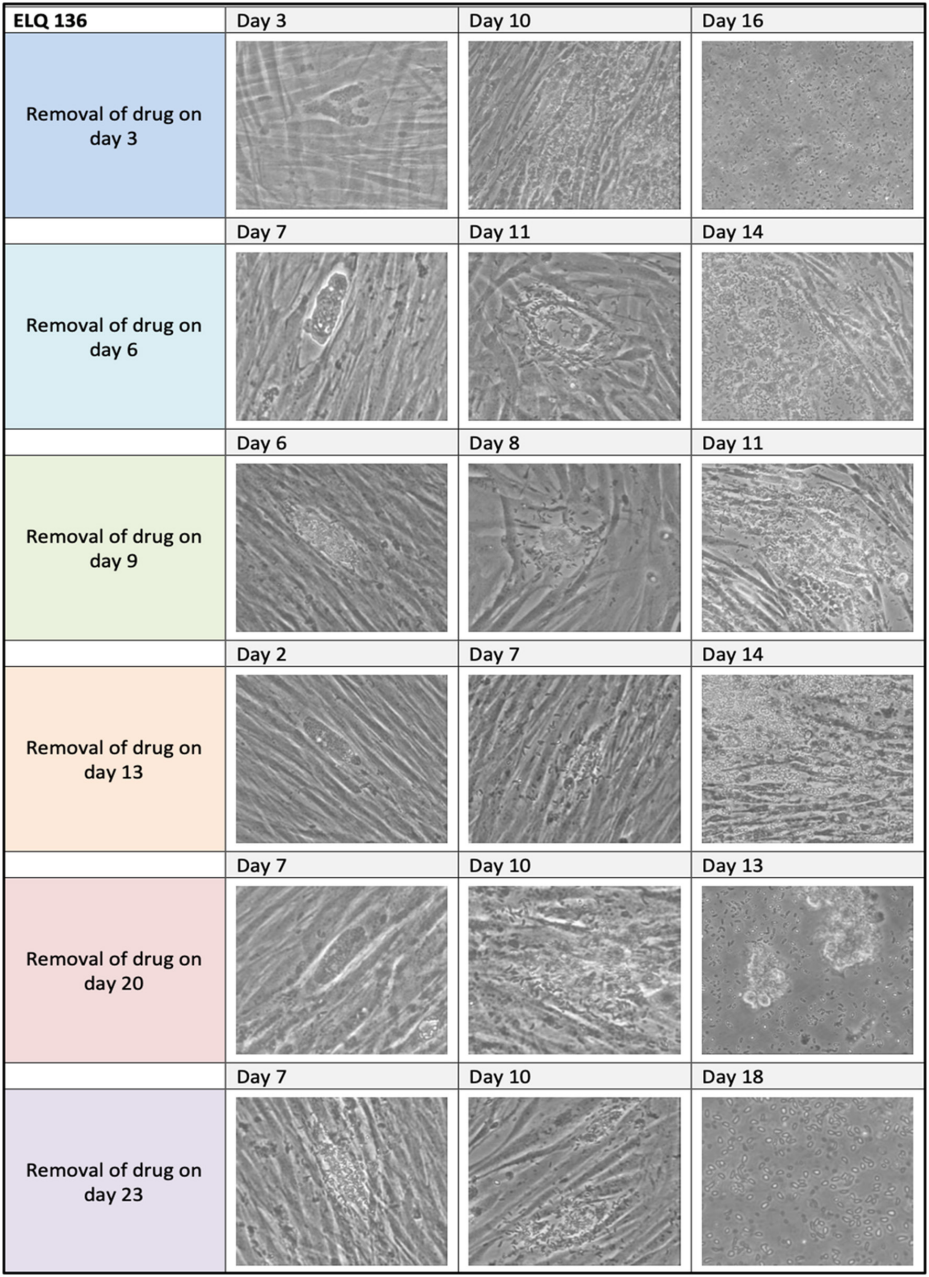
ELQ-136 long-term treatment assay. 2.5 μM of ELQ-136 was administered from 3 days until 23 days as indicated on the left. Drug pressure was released, and cells were kept in culture with medium for extended periods of time (3–18 days). Note that regrowth of parasites was seen in all cases.

Results on long-term treatment assays are documented for ELQ-121, ELQ-136 and -334 in Table 3, and are visualized for ELQ-136 in Figure 4. In the control cultures supplemented with the appropriate concentration of DMSO as a solvent control, *B. besnoiti* tachyzoites proliferated rapidly and the host cell layer was destroyed after 3–4 days post-infection. In ELQ treated cultures, parasite growth was much delayed, regardless which drug was used. Until day 3 of treatment, few large parasitophorous vacuoles developed. More often, small vacuoles could be observed and few extracellular parasites could be seen. Removing the drug pressure 3 days after a continued treatment, resulted in increased proliferation and finally complete lysis of the host cell layers within 17 days post-drug removal in the case of ELQ-121, 16 days for ELQ-136 (see Figure 4) and 10 days for ELQ-334. Continued treatment for 13 days resulted in lysis of the entire host cell monolayer at 21 days after drug removal for ELQ-121, at 14 days for ELQ-136 and at 10 days for ELQ-334. Even after the maximum period of treatment for 23 days, parasites recovered from the treatment and proliferated again after drug pressure removal, and complete host cell lysis was achieved 18 days after drug removal for ELQ-121, and 10 days for ELQ-136.

**Table 3:** Long term treatments with ELQ-121, ELQ-136 and ELQ-334 act parasitostatic.

**Compound**	**Treatment culture time (days)**	**Post-treatment culture until host cell lysis**
ELQ 121	3	17
	6	23
	9	23
	13	21
	20	18
	23	18
ELQ 136	3	16
	6	14
	9	11
	13	14
	20	13
	23	10
ELQ 334	3	10
	6	11
	9	11
	13	10
	20	ND
	23	ND

*T25 tissue culture flasks were infected with B. besnoiti tachyzoites, and the compounds were added all at 2.5 μM concentration. Culture continued for the time spans indicated, with medium changes and addition of fresh compounds every 3–4 days. The cultures were then washed with medium to remove the drugs and then incubated further in the absence of the compounds until host cell lysis occurred. ND, not done*.

**Figure 5:**
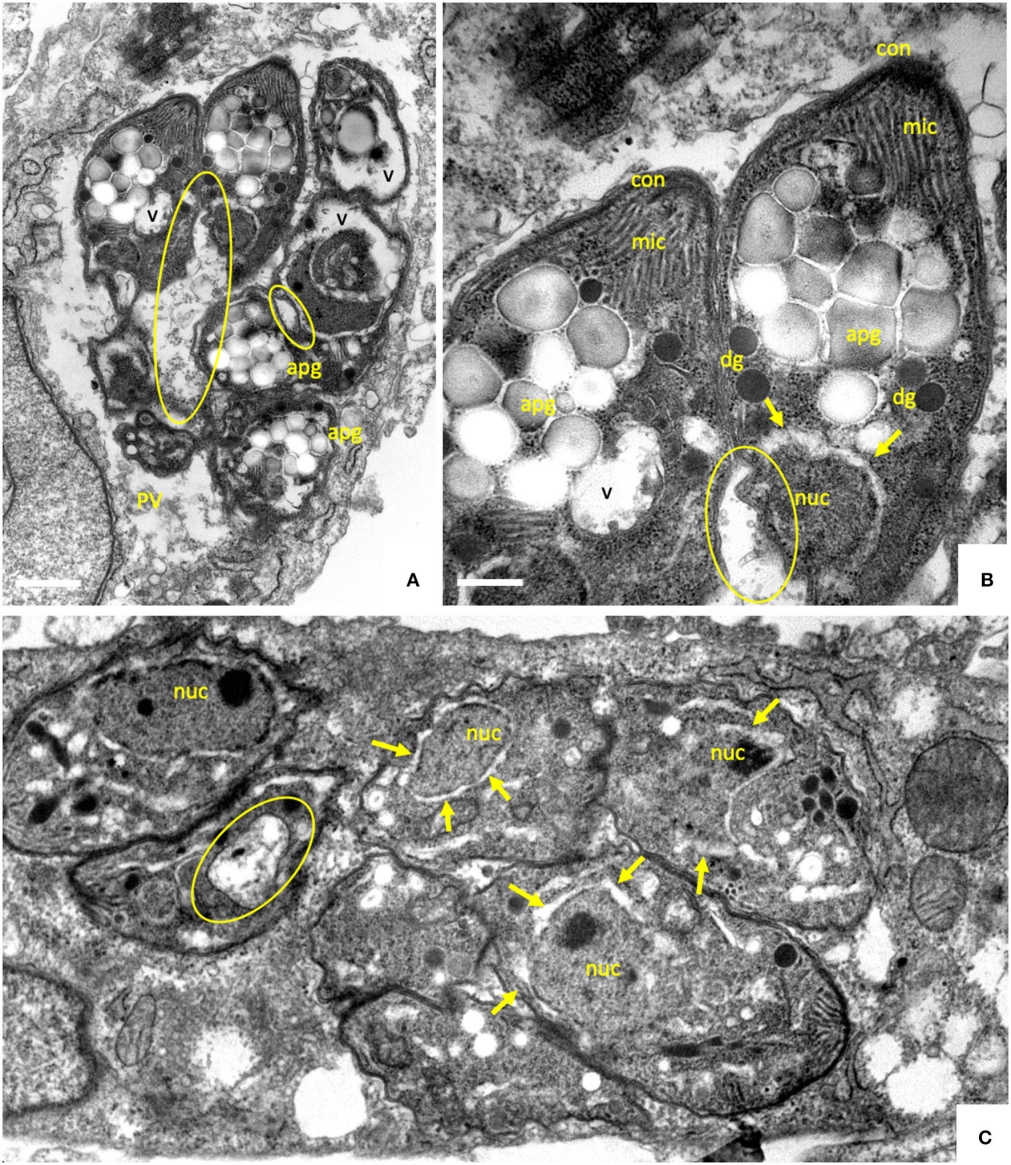
TEM of *B. besnoiti* tachyzoites treated with 2.5 μM of ELQ-316 during 20 days, and then cultured without drug during 10 days. The encircled areas indicate the mitochondrion, arrows point to a thickening of the nuclear membrane; nuc, nucleus; dg, dense granules; rhop, rhoptries; mic, micronemes; apg, amylopectin granules. Bars in **(A)** = 500 nm; **(B)** = 200 nm; **(C)** = 350 nm.

Thus, after an initial inhibition of proliferation, *B. besnoiti* tachyzoites exhibited the capacity to efficiently adapt to prolonged treatments at increased ELQ concentrations. In order to investigate whether this adaptation would also result in morphological or structural alterations, *B. besnoiti* cultures that had been treated with 2.5 μM ELQ-136 during 20 days and then further cultured in medium without drug, were subjected to TEM analysis (Figure 5). Many parasites in these cultures exhibited peculiar structural changes. A large portion of parasites formed large amorphous bodies within their cytoplasm, which closely resembled amylopectin granules (Figures 5A,B). Amylopectin granules are often seen in the bradyzoites of cyst-forming apicomplexans. In addition, major parts of the cytoplasm were vacuolized, and no intact mitochondrion could be detected in these parasites, indicating that these vacuoles were derived from degenerated mitochondria. Other parasitophorous vacuoles contained multiple parasites devoid of amylopectin granules (Figure 5C), but with altered thickened nuclear membrane and larger vacuoles, also most likely mitochondrial remnants. No intact mitochondrial structures could be visualized in these parasites.

## Discussion

We here report on the *in vitro* efficacy of distinct ELQ-analogs against *B. besnoiti* tachyzoites. ELQs act on the cytochrome *bc*_1_ complex. At the oxidative (Qo) site of cytochrome *b*, ubiquinol is oxidized to ubiquinone by releasing two electrons and two protons, and at the reductive (Qi) binding site, ubiquinone is reduced to ubiquinol (30). Blocking only one Q site, either Qo or Qi, results in an inhibition of the catalytic functioning of the complex, which leads to a collapse of the electrochemical gradient and oxidative phosphorylation, or in the case of *Plasmodium*, pyrimidine starvation due to a lack of ubiquinone for its biosynthesis. The scaffold of 4(1H)-quinolone is compatible with binding at either the Qo or Qi site and depending on the quinolone core substituents. Dual-site inhibition of cytochrome *b* is favorable in terms of resistance formation, as this would require two independent mutations within the same protein target appearing at the same time (31).

Fourteen ELQs with side chain variations coupled with Q-site targeting substituents were screened. These had been previously generated to test structure-activity relationships of Qi vs. Qo site selectivity in *Plasmodium* and *Toxoplasma* (20, 22). The compounds have different alterations, such as an alkyl or diphenyl ether side chain at the third position and several substitutions at positions 4, 5, 6, or 7 of the endochin ring.

The primary screening showed (i) that quinolones containing fluorine atoms at position 5 or 5,7 in combination with a 3-alkyl side chain are the most active compounds against *B. besnoiti* tachyzoites *in vitro*, and (ii) that *B. besnoiti* tachyzoites were generally less susceptible to ELQ treatment compared to the closely related *N. caninum* and *T. gondii*. Five compounds were selected for determination of IC50 values by quantitative real time PCR. In 3-days proliferation assays, ELQ-316 and its prodrug ELQ-334 inhibited *B. besnoiti* tachyzoite proliferation with IC50s of 7.97 and 5.81 nM, respectively. In comparison, the IC50 values against *N. caninum* tachyzoites for ELQ-316 and ELQ-334 were 0.66 and 3.33 nM, respectively. Oral administration of ELQ-334 led to improved pup survival and inhibition of vertical transmission in a pregnant neosporosis mouse model (24). The IC50 value of ELQ-316 against *T. gondii* tachyzoites was in the same range as *N. caninum* (0.35 nM), and experiments in murine toxoplasmosis models have shown that ELQ-316 was highly efficacious in acute infection mediated by *T. gondii* tachyzoites, and also reduced the tissue cysts burden *in vivo* in a latent toxoplasmosis infection model (18). The lowest IC50 values in *B. besnoiti* were demonstrated for ELQ-121 and ELQ-136, with 0.49 and 2.63 nM, respectively. Nevertheless, the IC50 of ELQ-121 was roughly 5 and 80 times higher than the reported IC50 values for *N. caninum* and *T. gondii*, and the IC50 of ELQ-136 was 130 and 20 times higher, respectively (22, 24).

Another compound of interest was ELQ-400, which was identified earlier as an active compound against *N. caninum* infection through a screening of the MMV (medicines for malaria venture) pathogen box (25). In *N. caninum*, ELQ-400 exhibited an IC50 value that was similar to ELQ 334, inhibited both host cell invasion and intracellular proliferation of *N. caninum* tachyzoites *in vitro*, and induced strong alterations in the parasite mitochondrion (25). However, ELQ-400 was more efficient than ELQ-334 in preventing cerebral infection in non-pregnant mice. The higher activity of ELQ-400 in *N. caninum*, in comparison to other ELQs, may be due to pharmacokinetics (21, 22). However, in *B. besnoiti*, ELQ-400 exhibited an *in vitro* IC50 of 72.2 nM, which is ~18 times higher than the reported value for *N. caninum*. Possible causes for the observed discrepancies in activities among the three apicomplexans could be differential uptake of drugs in different species, and off-target effects that occur in one species but not in the other. These effects may result in an additive or synergistic activity in concert with cytochrome *bc*_1_ inhibition (22, 29). In addition, the use of different isolates and host cell lines, culture conditions and methods of quantification could be attributed to different IC50 values.

Discrepancies in drug susceptibilities could potentially also be based on slight variations in the molecular target sequences that affect ELQ binding. In this respect, we identified no sequence divergence among *T. gondii* and *B. besnoiti* in the predicted Qo binding pockets and only 3 aa acid substitutions that differed in the *T. gondii* and *B. besnoiti* Qi sites, two in the D-Helix and one in the de Loop region of the Qi site. To what extent, these differences affect the interactions with the ELQs investigated here is not known. The different substitutions in the structure of the ELQs are responsible for the activity and the differential binding of the drug to either the Qi site, the Qo site, or both sites (20). Thus, SAR analysis showed that ELQ-121 and ELQ-136 target the cytochrome *b* Qo site whereas ELQ-316 targets the cytochrome *b* Qi site of the complex (22).

The almost complete identity of sequences of the Qi and Qo binding pockets of *T. gondii* and *B. besnoiti* indicates that ELQs target the cytochrome *bc*_1_ complex of *B. besnoiti*. TEM showed that during the treatment of *B. besnoiti* and *N. caninum* tachyzoites with ELQs, the parasite mitochondrion clearly lost its characteristic electron dense matrix with visible cristae. Interfering with the electron transport chain of the mitochondrion causes the release of electrons, which possibly can relocate to other biomolecules. This might result in an induction of free radicals and ROS, which are harmful for the parasite. Thus, it appears that a strategy of this parasite to avoid these adverse effects is to get rid of a structurally intact mitochondrion, and switch from oxidative phosphorylation to glycolysis. The intermediate metabolites needed for glycolysis would then have to be scavenged from the host cell.

Indeed, *B. besnoiti* tachyzoites exhibited structural changes in their mitochondrial matrix already after 6 h of treatment, and these changes became progressively more obvious at later timepoints for all compounds investigated. In addition to the mitochondrial alterations, TEM also detected a gradual thickening of the nuclear membrane, the integrity of which was altered in the drug-treated parasites compared to the untreated specimens as it appeared partially separated, creating a wider distance between nucleus and surrounding cytoplasm. Such a compartmentalization of the cytoplasm could possibly be an effect of autophagy upon chronic mitochondrial respiratory chain impairment (32). However, this needs to be further investigated. Also, while there is clear evidence that ELQs are targeting the ubiquinone binding site of the cytochrome *bc*_1_ complex, it should be considered, that there is also non-mitochondrial ubiquinol, and there are potential ELQ binding sites outside the mitochondrion that might be targeted by these compounds. For instance ubiquinone also contributes to electron transport as a transmembrane component of the Golgi apparatus (33).

While these changes within the first 48 h of drug treatment appeared dramatic, we found that *B. besnoiti* tachyzoites exhibited an outstanding adaptive potential, in that after continuous treatment for 20 days they had survived and resumed proliferation, albeit at a slower rate. Visualization of these parasites by TEM revealed that they had adopted profound structural alterations. In many instances, the cytoplasm of treated and recovered parasites was filled with round amorphous bodies that closely resembled amylopectin granules normally found in bradyzoites. Amylopectin granules were also identified in *B. besnoiti* tachyzoites treated with several bumped kinase inhibitors (BKIs) (11), nitazoxanide (9), pentamidine derivatives (10), and in *Besnoitia* treated with another cytochrome *bc*_1_ complex inhibitor, the naphtoquinone buparvaquone (13). Thus, these features are most likely associated with general physiological stress and potentially with metabolic adaptation rather than being specific indicators of the mechanism of action of these compounds. Interestingly, also upon prolonged buparvaquone treatment, *B. besnoiti* has been shown to adapt to increased concentrations of buparvaquone, and buparvaquone-adapted *B. besnoiti* also lacked a mitochondrion with an electron dense matrix and discernible cristae (13). It thus appears that a structurally intact mitochondrion is not a prerequisite for parasite survival, pointing toward alternative metabolic pathways beside oxidative phosphorylation for generating energy, such as glycolysis, and possibly increased scavenging of essential metabolites from the host cell.

In future investigations, drug combination approaches could be considered. A combination of the prodrug ELQ-334, a Qi site inhibitor, with a Qo site inhibitor may have higher activity and increased efficacy due to the dual site inhibition effect. This was achieved for the treatment of *Babesia microti*, where a combination of ELQ-334 and atovaquone resulted in a radical cure (31). The availability of well-established and standardized experimental *in vivo* models for bovine besnoitiosis would be an important prerequisite to carry out such studies in the target host (2).

## Data Availability Statement

The raw data supporting the conclusions of this article will be made available by the authors, without undue reservation, to any qualified researcher.

## Author Contributions

AH and JD conceived and designed the study. AL and SF established the Bb Lisbon isolate and guidance on cell culture, and AH coordinated the biological assays part of the study. NE, VB, JM, and NM performed cell culture, *in vitro* assays and inhibition studies. NE and VB performed real time PCR, and TEM studies were carried out by NE and AH. SP, AN, MR, RW, and JD designed, synthesized, and provided ELQ compounds. NE and AH did the interpretation of results, and NE, AH, AL, and JD wrote the manuscript.

### Conflict of Interest

The authors declare that the research was conducted in the absence of any commercial or financial relationships that could be construed as a potential conflict of interest.
